# Cooperative Reinforcement of Ionic Liquid and Reactive Solvent on Enzymatic Synthesis of Caffeic Acid Phenethyl Ester as an In Vitro Inhibitor of Plant Pathogenic Bacteria

**DOI:** 10.3390/molecules22010072

**Published:** 2017-01-02

**Authors:** Yan Xu, Sheng Sheng, Xi Liu, Chao Wang, Wei Xiao, Jun Wang, Fu-An Wu

**Affiliations:** 1 School of Biotechnology, Jiangsu University of Science and Technology, Zhenjiang 212018, China; xuyan@just.edu.cn (Y.X.); shengsheng@just.edu.cn (S.S.); 18260633007@163.com (X.L.); ktwangchao@126.com (C.W.); 15262916031@163.com (W.X.); 2 Sericultural Research Institute, Chinese Academy of Agricultural Sciences, Zhenjiang 212018, China

**Keywords:** biocatalysis, ionic liquid, caffeic acid phenethyl ester, reactive solvent, *Ralstonia solanacearum*, antibacterial activity

## Abstract

It is widely believed that lipases in ionic liquids (ILs) possess higher enzyme activity, stability and selectivity; however, reaction equilibrium is always limited by product inhibition, and the product is difficult to separate from non-volatile ILs using distillation. To solve this problem, using trialkylphosphine oxide (TOPO) as a complexing agent, a novel biphase of reactive solvent and IL was firstly reported for caffeic acid phenethyl ester (CAPE) production from methyl caffeate (MC) and 2-phenylethanol (PE) catalyzed by lipase via transesterification. The effects of the reaction parameters and their action mechanism were investigated, and the inhibition of CAPE against bacterial wilt pathogen *Ralstonia solanacearum* was firstly measured. The MC conversion of 98.83% ± 0.76% and CAPE yield of 96.29% ± 0.07% were obtained by response surface methodology in the 25 g/L TOPO-cyclohexane/[Bmim][Tf_2_N] (1:1, *v*/*v*); the complex stoichiometry calculation and FTIR spectrum confirmed that the reversible hydrogen-bond complexation between TOPO and caffeates significantly enhances the cooperative effect of two phases on the lipase-catalyzed reaction. The temperature was reduced by 14 °C; the MC concentration increased by 3.33-fold; the ratio of catalyst to donor decreased by 4.5-fold; and *K*m decreased 1.08-fold. The EC_50_ of CAPE against *R. solanacearum* was 0.17–0.75 mg/mL, suggesting that CAPE is a potential in vitro inhibitor of plant pathogenic bacteria.

## 1. Introduction

Biphase catalysis is an important protocol in a number of synthesis processes and has been identified as an active technique in many chemical reactions [1,2,3]. This is because homogeneous catalysis is usually accompanied with the obvious drawbacks of laborious separation, product inhibition and recycling of catalysts [4]. Specifically, a biphase system, as a versatile reaction medium, has attracted significant attention for different types of biocatalysis over the past ten years [5], which is regarded as a practical alternative to traditional chemical synthesis [6]. In general, an aqueous/organic biphasic system has many applications due to the fact that the biocatalyst locates in a “mobile phase” [7], and the reaction takes place in the aqueous phase or at the surface boundary between phases [1]. After the reaction, the catalyst can be separated from the products via a simple phase separating. However, an aqueous/organic system is not suitable for water-sensitive lipase-catalyzed reactions. Recently, ionic liquids (ILs) have been applied as solvents in lipase-catalyzed reactions to synthetize a myriad of products [8,9,10]. It is widely believed that lipases in ILs possess higher activity, stability and selectivity, which can increase the yield of products and the conversion of feedstocks [11]. However, reaction equilibrium is always limited by product inhibition, and the product is difficult to separate from the non-volatile ILs using distillation [12]. To solve this problem, some approaches have been attempted to form a biphase with other solvents, such as a fluorous biphasic system [13], supercritical CO_2_ [14], poly(ethylene glycol) linked dicationic acidic IL-toluene [7], etc. Thus, it is highly desirable to develop a novel biphase system containing ILs and solvents with high efficiency for the lipase-catalyzed reaction.

Caffeic acid phenethyl ester (CAPE) is a high-value chemical compound firstly isolated from the honeybee propolis, which has various biological and pharmacological activities [9]. Its isolation from natural resources is inefficient, time-consuming and uneconomical. CAPE is historically synthesized via chemical routes, which are neither efficient, nor environmentally friendly [15,16]. Lipase-catalyzed CAPE synthesis has been regarded as a promising approach due to its high specificity and mild conditions (environmentally friendly, as well), but is also challenging. Actually, most of the pioneering publications reported that the CAPE yields generated from caffeic acid (CA) and 2-phenylethanol (PE) via esterification catalyzed by Novozym 435 in isooctane at about 70 °C were unstable, although their CA conversions were above 90% [17,18]. Because CAPE is extraordinarily active, readily degraded or transformed into other by-products, particularly at high temperatures [11], therefore IL is a “greener” medium instead of conventional solvent and prevented the drawbacks of effumability, lipase inhibition and product instability. Using ILs containing anion [Tf_2_N]^−^ (i.e., 1-ethyl-3-methylimidazolium bis(trifluoromethyl)sulfonyl]imide ([Emim][Tf_2_N]) and 1-*n*-butyl 3-methylimidazolium bis(trifluoromethylsulfonyl)imide ([Bmim][Tf_2_N])) as a medium, CA conversion was obtained from 92% [8] to 98.75% [11], but the CAPE yield is still only approximately 64.55%. To solve this problem, our preliminary study proposed two schemes: one is to add 2% (*v*/*v*) DMSO as a co-solvent in the IL to improve the solubility of CA from 4.5 g/L–9 g/L, obtaining a CAPE yield of 79.53% via esterification from CA and PE [19]; the other is to use a continuous-flow packed-bed microreactor to replace a batch reactor, using methyl caffeate (MC) to replace CA, and a CAPE yield of 93.21% was achieved via transesterification from MC and PE [9]. Although both schemes could improve the synthesis efficiency and obtain a high substrate conversion, the results are still not satisfactory due to low yield and the requirement of special equipment. Since the lipase-catalyzed synthesis of CAPE via transesterification using MC as a simple and accessible feedstock has been proven a feasible procedure [20,21], [Bmim][Tf_2_N] is suitable for the dissolution of substrate MC due to its polarity being lower than that of [Emim][Tf_2_N] [22], so we proposed a novel biphase of reactive solvent/[Bmim][Tf_2_N] based on the idea of improving substrate solubility and the principle of biphase catalysis. In the proposed scheme, a complexing agent was added to enhance the enzymatic synthesis of CAPE from MC and PE via transesterification.

**Figure 1:**
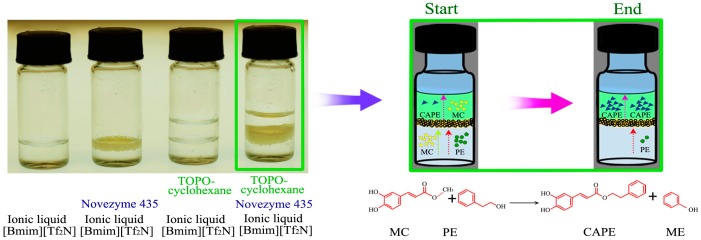
A biphase system of trialkylphosphine oxide (TOPO)-cyclohexane/[Bmim][Tf_2_N] for lipase-catalyzed synthesis of caffeic acid phenethyl ester (CAPE) from methyl caffeate (MC) and 2-phenylethanol (PE) via transesterification.

In the past few decades, complexation extraction has shown promise as a conventional and effective technology for the separation and recovery of the polar organic species from diluents with high efficiency and selectivity in chemical industry [23]. Most extractive recovery of solutes from dilute aqueous solutions used neutral or acidic donor ligands, such as tri-*n*-octylphosphine oxide (TOPO), tri-*n*-butyl phosphate (TBP) and carbamoylmethylphosphine oxide (CMPO) and di-2-ethylhexylphosphoric acid (D_2_EHPA). TOPO has been used as a complexing agent in a reactive extraction method to recover mono-caffeoylquinic acids (mono-CQAs, containing one caffeoyl group) from tobacco waste [24]. Furthermore, TOPO dissolved in nonpolar solvents can be mixed with the conventional aqueous solutions or nonconventional solvents like ILs to form a biphasic system for the lipase-catalyzed reaction [25]. In our preliminary study, we have demonstrated that a complex extraction system consisting of TOPO-solvent and IL could sufficiently recover CAPE [9]. However, to the best of our knowledge, no report has addressed the use of TOPO in TOPO-cyclohexane/[Bmim][Tf_2_N] to intensify the enzymatic synthesis (see Figure 1), the enzymatic catalysis theory and its application of the novel biphasic system require further research.

CA and its esters exhibit promising antimicrobial activities by attacking RNA, DNA and protein, preventing bacterial translocation [26,27]. The antibacterial effect of CAPE has been reported on oral cancer cells [28], leukemia HL-60 cells [29], Gram-positive and -negative bacteria [30], *Helicobacter pylori* [31], etc. However, there is no report of the antibacterial activity of CAPE against *Ralstonia solanacearum*, which causes serious bacterial wilt in the world [32]. Bacterial wilt caused by *R. solanacearum* is one of the most widely infectious and damaging plant diseases [33], which affects approximately 300 species across 50 families, including herbs, shrubs and trees [34]. It has been difficult to control bacterial wilt, and the conventionally chemical control methods, such as 2% methanol and 5% whitewash, are harmful to the environment. Therefore, the anti-pathogen activity of our lipase-catalyzed CAPE was also studied in this work.

The aim of this work was to develop a novel biphasic system for highly efficient CAPE synthesis. For this purpose, the effect of TOPO concentration, substrate concentration, substrate molar ratio, reaction temperature and lipase usage on the MC conversion and CAPE yield, as well as the stability of the system were investigated in the biphase system of TOPO-cyclohexane/[Bmim][Tf_2_N]. Furthermore, kinetic models for the lipase-catalyzed synthesis of CAPE using different media were proposed and compared, the secondary structure change of the lipase and complex characteristic between TOPO and caffeates measured by FTIR spectrometry. Additionally, the growth inhibition activity of CAPE against bacterial wilt pathogen strain *R. solanacearum* was measured.

## 2. Results and Discussion

### 2.1. Selective Complexation of TOPO and Caffeates in Cyclohexane/[Bmim][Tf_2_N]

**Figure 2:**
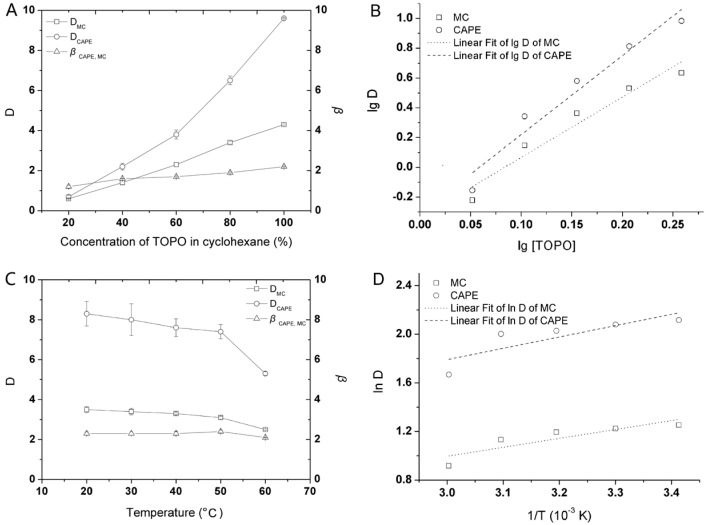
Effect of TOPO concentrations in equivoluminal cyclohexane on the complex formation of TOPO and caffeates from lipase-catalyzed transesterification reactants in [Bmim][Tf_2_N] (**A**) and lg *D*-lg [TOPO] curves of caffeates in the presence of TOPO (**B**); the effect of temperature on the complex formation of TOPO and caffeates from lipase-catalyzed transesterification reactants in [Bmim][Tf_2_N] using equivoluminal TOPO-cyclohexane (**C**) and lg *D*-1/*T* curves of caffeates in the presence of TOPO (**D**). Each value is expressed as the mean ± standard deviation (*n* = 3).

Figure 2A shows the complexing behaviors of CAPE and MC in the cyclohexane with TOPO ranging from 20 g/L–100 g/L (≈ saturation concentration). In total, the distribution coefficient (*D*) values of caffeates (including CAPE and MC) and the separation factor (β) values of CAPE vs. MC slightly increased with increasing concentration of TOPO over the range of 20–100 g/L. However, with a TOPO concentration greater than 60 g/L, the *D* values of CAPE began to quickly increase. Actually, when a 1:1 ratio of 100 g/L TOPO-cyclohexane to sample volume was used, the *D* values of CAPE and MC were 9.6 and 4.3, respectively, and the β value of CAPE vs. MC was 2.2. The results indicated that TOPO has a strongly and selectively complexing ability with caffeates in the biphase, and its complex formation is favored to CAPE, which also means the designed complexing solvents can be effectively used for the selective extraction of CAPE generated from the lipase-catalyzed transesterification. This is because TOPO is a neutral phosphorus extractant that contains one hydrophilic phosphoryl oxygen head group (P=O) and three medium chain alkyl groups (R = *n*-hexyl), with characteristics similar to those of non-ionic surfactants [35]. TOPO can exist at the liquid–liquid interface and form a monolayer where the P=O group points toward the IL phase and the hexyl chains point toward the organic phase. The specific chemical interaction between the complexing agent and caffeates to form a complex in the organic phase allows more caffeates to be extracted from the IL phase.

Usually, the stoichiometry of TOPO-caffeate complex formation in the organic phase was determined by the reactive extraction equilibrium, which could be written as a 1:*n* (caffeate:TOPO) complex [24]. The stoichiometry of the complex formation of TOPO and caffeates can be expressed as Equation (1):

(1) $$\mathrm{lg}D=n\;{\mathrm{lg}[\mathrm{TOPO}]}_{\mathrm{org}}\;+\;a$$

where *a* is a numerical constant and subscript _org_ is the species in the organic phase. The constant *n* was obtained by experimental data using linear fitting regression.

Figure 2B shows lg *D*-lg [TOPO] curves of caffeates in the presence of TOPO; the values of *n* for MC and CAPE were four (R = 0.9637) and five (R = 0.9685), respectively. The complex stoichiometry between TOPO and CAPE is 1:5, the value of which is significantly larger than that for MC (1:4), which indicates that the complex of TOPO-CAPE is more stable than the other complex of TOPO-MC. This is because organic chemicals containing the phenolic hydroxyl group on the benzene ring [11], including MC and CAPE, exist as dimers in an organic phase, especially in non-polar solvents, such as cyclohexane [36], as a result of strong intermolecular hydrogen bonding between four or five TOPO molecules and one caffeate molecule. 

Figure 2C shows the effect of temperature on the complex formation of TOPO and caffeates from lipase-catalyzed reactants in [Bmim][Tf_2_N] using equivoluminal TOPO-cyclohexane; the *D* values of CAPE and MC decreased with increasing temperature from 20 °C–60 °C, which indicated that the complex formation and selective extraction of CAPE in the presence of TOPO is an exothermic process. However, the β value of CAPE vs. MC decreased very slightly between 20 and 60 °C, which dropped from 2.3 to just 2.1. Therefore, there is a very small effect of temperature on the complex formation of TOPO and caffeates from the enzymatic reactants in the cyclohexane.

In addition, following the Clausius-Clapeyron equilibrium equations, the relationship between *D* and isosteric extraction enthalpy Δ*H* (kJ/mol) can be expressed as Equation (2) in natural-logarithm form:

(2) lnD=lnb − ΔH/(RT)

where *T* is absolute temperature (K), *R* is ideal gas constant and b is a numerical constant.

Figure 2D shows the lg *D*-1/*T* curves of caffeates in the presence of TOPO, the isosteres of which form straight lines with correlation coefficients *R* greater than 0.8390. The results also indicated that re-extraction of CAPE from the complexing solvents of TOPO-cyclohexane to aqueous solution could be easy due to the complex formation always being released at high temperatures [23]. Thus, from the theoretical point of view, the biphase of TOPO-cyclohexane/[Bmim][Tf_2_N] is good for reversible complexation of TOPO and caffeates, to increase the substrate concentration when the enzymatic reaction is beginning, to weaken the product reflection when the reaction is continuous, also to simplify the purification procedure of CAPE with a high concentration using the re-extraction technique at a higher temperature when the reaction is finished.

**Figure 3:**
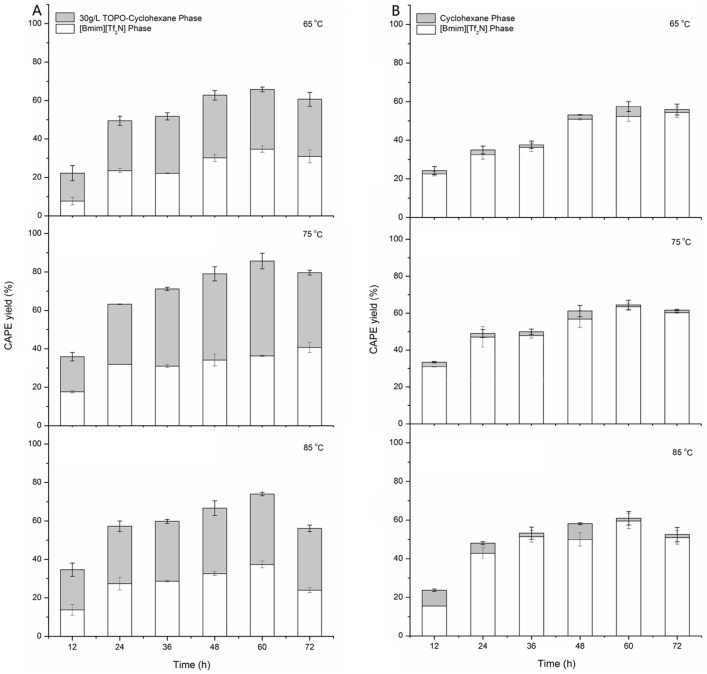
Comparison of CAPE yield from MC and PE via transesterification using equivoluminal 30 g/L TOPO-cyclohexane/[Bmim][Tf_2_N] (**A**) and cyclohexane/[Bmim][Tf_2_N] (**B**) at different temperatures. The upper histogram shows the yield of CAPE in the solvent phase; the lower histogram shows the yield of CAPE in the ionic liquid (IL phase); and the total CAPE yield is the sum of the upper and lower yields. Conditions: mass ratio of MC to Novozym 435 = 1:20, molar ratio of MC to PE = 1:15, MC concentration = 10 g/L, 180 rpm for 72 h. Each value is expressed as the mean ± standard deviation (*n* = 3).

Figure 3 shows that after adding 30 g/L TOPO in the biphase of cyclohexane/[Bmim][Tf_2_N], the total CAPE yields at 65 °C, 75 °C and 85 °C were 65.77%, 85.67% and 73.97%, increasing by 14.52%, 32.99% and 21.40%, respectively. The result indicated that the addition of TOPO in cyclohexane plays an important role in improving the synthesis efficiency of CAPE. Thus, TOPO-cyclohexane/[Bmim][Tf_2_N] as a novel medium for the lipase-catalyzed synthesis of CAPE from MC and PE via transesterification has a significant technical feasibility and needs to be optimized from an engineering perspective.

### 2.2. Effect of TOPO Concentration in Cyclohexane

**Figure 4:**
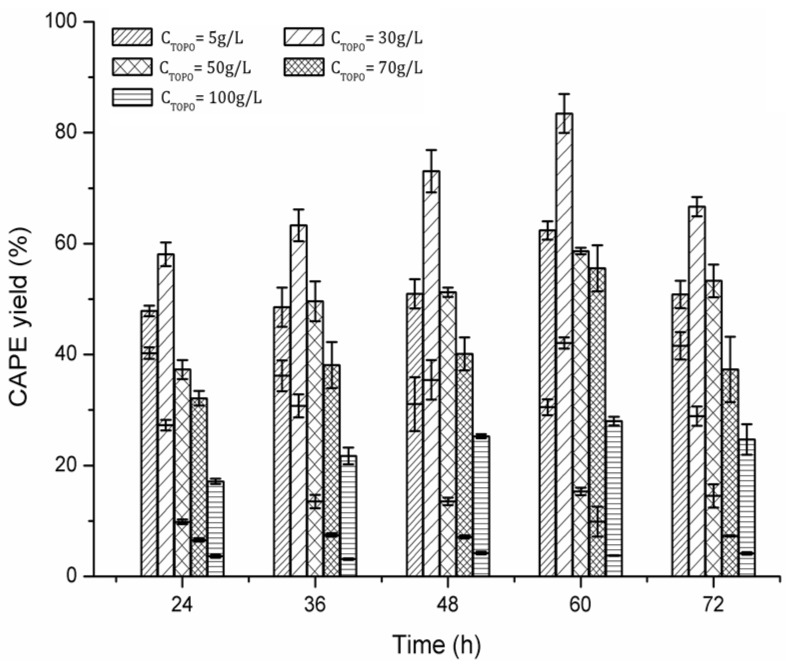
Effect of TOPO concentration in cyclohexane on the yield of CAPE in TOPO-cyclohexane/[Bmim][Tf_2_N] (1:1, *v*/*v*). The upper histogram shows the yield of CAPE in TOPO-cyclohexane phase; the lower histogram shows the yield of CAPE in the IL phase; and the total CAPE yield is the sum of upper and lower yields. Conditions: mass ratio of MC to Novozym 435 = 1:20, molar ratio of MC to PE = 1:15, MC concentration = 10 g/L, temperature = 80 °C. Each value is expressed as the mean ± standard deviation (*n* = 3).

Figure 4 shows the effect of TOPO concentration on the yield of CAPE in the TOPO-cyclohexane/[Bmim][Tf_2_N] (1:1, *v*/*v*) system at 80 °C. When the TOPO concentration in cyclohexane was 30 g/L, the total CAPE yield of 85.67% was obtained at 60 h; however, further increases in the TOPO concentration of 30–100 g/L decrease the total CAPE yield. In addition, the proportional value of upper and lower yields also has a significant change. For an increase in the reaction time from 24–60 h, the CAPE molecules were preferably stored in the lower phase with a low concentration of TOPO (i.e., 5%); further increases in the TOPO concentration of 30–100 g/L led to a relatively stable proportional value of upper and lower yields. The result also confirmed that the complex stoichiometry between TOPO and CAPE is greater than that between TOPO and MC. However, when the reaction time excessed 60 h, the total CAPE yields began to decrease, and the proportional value of upper and lower yields reverted at 24 h. This also confirms that CAPE was degraded or transformed into other by-products after a long time at high temperatures [11].

**Figure 5:**
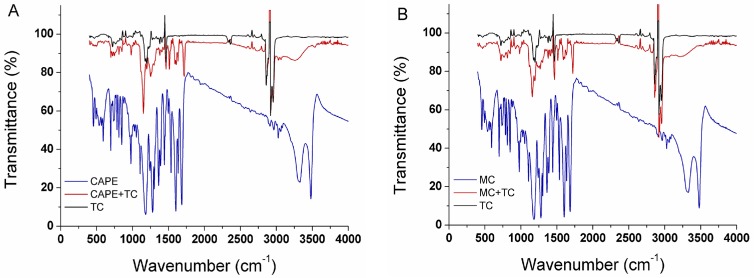
IR spectra of TOPO before and after complexation with CAPE (**A**) and MC (**B**) in 30 g/L TOPO-cyclohexane (TC)/[Bmim][Tf_2_N] (1:1, *v*/*v*).

To further confirm the complex formations between caffeates and TOPO in the cyclohexane, the resulting products obtained using the established complexation method were analyzed by FTIR. Figure 5 gives the IR spectra of TOPO before and after complexation with CAPE (A) and MC (B) in the 30 g/L TOPO-cyclohexane (TC)/[Bmim][Tf_2_N] (1:1, *v*/*v*) system. In the IR spectrum of pure TOPO in cyclohexane, the 2860–2955 cm^−1^ position denotes the C-H stretching vibration; the 1465 cm^−1^ position denotes the C-H asymmetric deformation vibration; the 1378 cm^−1^ position denotes the C-H symmetric deformation vibration; the 721 cm^−1^ position denotes the CH_2_ phase rock vibration of (CH_2_)_7_; the 1176 cm^−1^ position denotes the P=O stretching vibration; and the 1407 cm^−1^ position denotes the P-C deformation vibration [24]. These results demonstrate the existence of strong intermolecular interactions among TOPO molecules. A comparison of the IR spectra of pure CAPE and MC reveals that both caffeates are very similar. In the IR plots of pure CAPE and MC, the peaks at 1602 cm^−1^, 1496 cm^−1^ and 1442 cm^−1^ indicate the existence of a benzene-related structure, and the 1684 cm^−1^ and 3326 cm^−1^ peaks denote the existence of –COO^−^ and -Ph-OH groups, respectively. After being complexed with CAPE or MC, the P=O stretching vibration peak moved significantly; the wavenumbers of the peaks are presented in Table 1. After TOPO was complexed with MC and CAPE, the wavenumbers of the P=O stretching vibration peak shifted by 19 cm^−1^ and 25 cm^−1^, respectively, i.e., the greater the acid strength, the greater the change. These kinds of interactions result in the P=O stretching vibration position being moved to lower wavenumbers, and their bond strength decreased significantly. These two characteristics both indicated that the existence of a strong intermolecular interaction arises from the hydrogen bond between the O atoms in the P=O groups in TOPO and the H atoms in the CAPE. On the contrary, for MC and CAPE, these complexation interactions also result in the infrared sorption strength being significantly decreased and in the positions of the –COO^−^ and –Ph-OH groups’ vibration being moved to lower wavenumbers by 52 cm^−1^ and 53 cm^−1^, 16 cm^−1^ and 65 cm^−1^, respectively. Thus, TOPO in the cyclohexane with a suitable concentration could be well complexed with MC and CAPE from the reactants and could enhance the enzymatic synthesis of CAPE at the bottom.

### 2.3. CAPE Synthesis from MC and PE Using the TOPO-Cyclohexane/[Bmim][Tf_2_N] System

**Figure 6:**
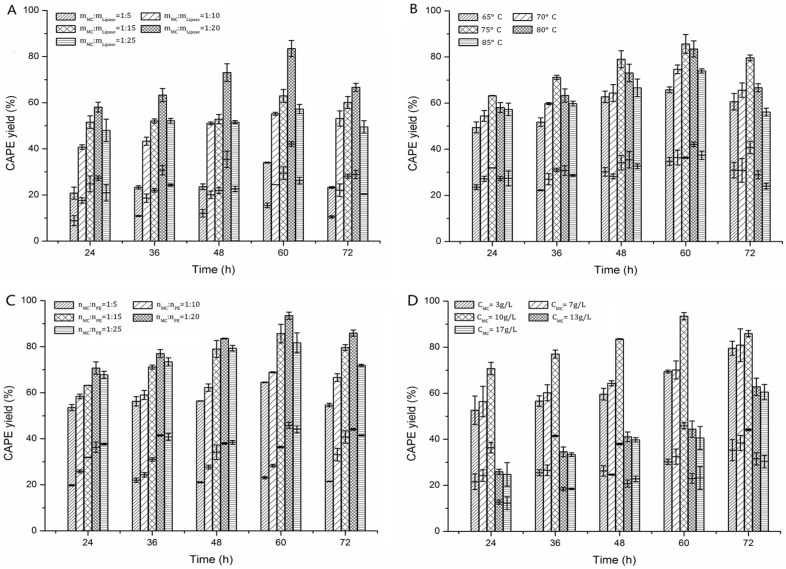
Effects of the mass ratio of MC to Novozym 435 (**A**), temperature (**B**), the molar ratio of MC to PE (**C**) and the MC concentration (**D**) on the yield of CAPE in the TOPO-cyclohexane/[Bmim][Tf_2_N] (1:1, *v*/*v*), respectively. The upper histogram shows the yield of CAPE in the TOPO-cyclohexane phase; the lower histogram shows the yield of CAPE in IL phase; and the total CAPE yield is the sum of upper and lower yields. Conditions: (A) molar ratio of MC to PE = 1:15, MC concentration = 10 g/L, temperature = 80 °C; (B) mass ratio of MC to Novozym 435 = 1:20, molar ratio of MC to PE = 1:15, MC concentration = 10 g/L; (C) mass ratio of MC to Novozym 435 = 1:20, MC concentration = 10 g/L, temperature = 75 °C; (D) mass ratio of MC to Novozym 435 = 1:20, molar ratio of MC to PE = 1:20, temperature = 75 °C. Each value is expressed as the mean ± standard deviation (*n* = 3).

Figure 6A shows the effect of lipase dosage on the yield of CAPE in 30 g/L TOPO-cyclohexane/[Bmim][Tf_2_N] (1:1, *v*/*v*) at 80 °C. The highest yield of CAPE is 83.47% at 60 h, with a mass ratio of MC to Novozym 435 as 1:20, beyond which there was a marginal decrease in yield. Hence, the amount of enzyme affected the reaction rate, which might be an outcome of the aggregation of the enzyme at high concentration, reducing the accessibility of enzyme particles to reactants [18,37]. In addition, the proportional value of upper and lower yields was always stable during the past 72 h, which indicated that the biphasic system led to a relatively stable synthesis of CAPE with a high yield.

Figure 6B shows the effect of reaction temperature on the yield of CAPE in the biphasic system; an increase in the reaction temperature during the range of 65–75 °C led to an increase of the total CAPE yield until 60 h. This indicated that the increasing of temperature might improve the solubility of MC in [Bmim][Tf_2_N], reduce IL viscosity and decrease mass transfer limitations [8,11]; also, this could strengthen the solubility of CAPE in solvents, accelerate the complexation reaction and promote the equilibrium of complex formation with TOPO. However, with a temperature higher than 75 °C, the total yield notably decreased, most likely due to partial damage on the complex formations between CAPE and TOPO and thermal inactivation of the enzyme. In total, total CAPE yield in 30 g/L TOPO-cyclohexane/[Bmim][Tf_2_N] at 75 °C was obtained after 60 h with 85.67%, and the proportional value of upper and lower yields also exerted a steady state during the past 72 h. In addition, Figure 3A shows that the CAPE yield in TOPO-cyclohexane was almost equal to or slightly higher than that in [Bmim][Tf_2_N]; however, Figure 3B shows that the CAPE yield in cyclohexane was absolutely lower than that in [Bmim][Tf_2_N]. It also confirms that temperature affects both the solubility of reactants and the reaction rate, as well as the solubility and formation rate of the complex between TOPO and caffeates.

Figure 6C shows the effect of the molar ratio of MC to PE on the yield of CAPE in the biphasic system. When the molar ratio of MC to PE was 1:20, the highest CAPE yield of 91.59% was obtained. Further increases in the molar ratio of MC to PE led to a slight decrease in the total CAPE yield. This dependency clearly implies enzyme inhibition by PE [19]. This inhibition could be due to the production of a dead-end inhibition complex by PE with the lipase, as described for the transesterification of 1-propanol with MC [38]. In addition, an increase in the molar ratio of MC to PE from 1:5–1:25 led to an increase of the CAPE yield in the lower phase because too much PE increased the solubility of substrate in the [Bmim][Tf_2_N] and changed the *D* values of caffeates between the two phases. Thus, a suitable excess of PE appears to be beneficial to biphase catalysis, perhaps because the complex formation of MC and TOPO is more easily decomplexed and releases MC to make contact with lipase and then generate the reaction.

Figure 6D shows the effect of MC concentration on the yield of CAPE in the biphasic system. When the range of MC concentration was 3–10 g/L, the CAPE yield of 79.55%–93.46% was obtained. Further increases in MC concentration result in a slight decrease in the total CAPE yield. Compared with the same reaction in [Bmim][Tf_2_N] [9], the suitable MC concentration improved from 3 g/L–10 g/L. Therefore, it can be concluded that the biphase system improved not only the enzyme enantioselectivity, but also the maximal substrate concentration as compared with the single phase [1]. Considering that the enzymatic synthesis of CAPE is of great interest for preparative purpose, there is a need to increase the substrate concentration, so the MC concentration should be as high as possible; however, a high CAPE yield and a good MC conversion should be maintained. Thus, the MC concentration in the biphasic system was selected as 10 g/L.

A Box-Behnken design (BBD) was employed to obtain the optimum conditions by the response surface methodology (RSM) (see the Electronic Supporting Information), for which the MC concentration was 10 g/L and the molar ratio of PE to MC was 20:1. Figure S1 shows that the optimal conditions for CAPE synthesis estimated by the model equation were as follows: temperature of 76 °C, TOPO concentration of 25 g/L and time of 59 h. Under the best conditions, the theoretical and experimental CAPE yields were 95.33% and 96.29% ± 0.07%, respectively.

### 2.4. Enzymatic Kinetics and Secondary Structure Change in TOPO-Cyclohexane/[Bmim][Tf_2_N]

The Michaelis-Menten model was used for the determination of the enzymatic kinetics in a batch reactor [9]. Figure S2 shows that the Lineweaver–Burk plots were linear. The kinetic constants and the corresponding correlation coefficient (R^2^) are listed in Table 2. The Michaelis constant *K*_m(app)_ value using 30 g/L TOPO-cyclohexane/[Bmim][Tf_2_N] (1:1, *v*/*v*) was 1.35 mM, which is 0.92-fold and 0.36-fold of that using [Bmim][Tf_2_N] (1.46 mM) and cyclohexane/[Bmim][Tf_2_N] (1:1, *v*/*v*) (3.75 mM), respectively. The lowest *K*_m(app)_ value shows the best performance of the combination of lipase and MC using 30 g/L TOPO-cyclohexane/[Bmim][Tf_2_N] (1:1, *v*/*v*). The formation of an enzyme-substrate complex is easier with the immobilized lipase due to the porous structure of the micro pipeline [19]. The improved kinetics might be attributed to better mass transfer in the biphasic system [1,5].

The secondary structure of lipase can be analyzed by FTIR since proteins absorb infrared wavelengths due to the peptide bond vibrations [39]. The amide I region (mainly due to the C=O stretching vibration) at approximately 1600–1700 cm^−1^ is mostly used in protein secondary structure determination due to its sensitivity in conformational changes [40]. Figure S3 shows that the resin influence of Novozym 435 on lipase can be eliminated due to the lack of the amide I region. Figure S4 shows the IR spectrum of lipase extracted from Novozym 435 used in different media; corresponding data calculated and transformed to secondary structure contents are listed in Table 3. The α-helix and β-sheet contents of the original Novozym 435 were 12.8% and 55.2%, respectively. A very high similarity has been found between the secondary structure of lipase used in the [Bmim][Tf_2_N] and 30 g/L TOPO-cyclohexane/[Bmim][Tf_2_N] (1:1, *v*/*v*), with the increase of reaction time; the α-helix contents both increased to the maximum before 12 h and then decreased; however, the β-sheet contents decrease at the first 36 h, then a small amount of increase occurred. However, using the cyclohexane/[Bmim][Tf_2_N] (1:1, *v*/*v*), the α-helix content decreased very slowly, but the β-sheet content reduced sharply. This shows that TOPO contributes to maintaining the enzymatic activity of lipase in cyclohexane.

In total, the decreases of α-helix and β-sheet contents significantly affect the active site of lipase [41]. Partially, the lower the α-helix and β-sheet contents, the easier the substrates access the active site [42]. It is reasonable to expect that more than one type of interaction must be broken before full enzyme deactivation occurs, followed by different changes of the native structure [43]. Thus, TOPO in cyclohexane attributes to maintaining the nature of IL, which results in a higher yield of CAPE.

### 2.5. Comparative Analysis of CAPE Synthesis Using Different Media

Under the optimal condition, the MC conversion of 98.83% ± 0.76% and CAPE yield of 96.29% ± 0.07% were obtained from MC and PE using 25 g/L TOPO-cyclohexane/[Bmim][Tf_2_N] (1:1, *v*/*v*) as the medium, which are the highest values of CAPE synthesis in the present reports. The results indicated that the yield of CAPE using lipase-catalyzed transesterification in a batch biphase system is higher than that using lipase-catalyzed esterification in a batch reactor [11,19] and using lipase-catalyzed transesterification in the packed bed microreactor [9].

In addition, the temperature was decreased from 90 °C down to 76 °C, in the biphase medium with TOPO to achieve the highest yield of CAPE in a batch reaction, while the reaction time was shortened from 72 h down to 60 h; *K*_m_ value decreased 1.08-fold, and the secondary structure changes confirmed that the lipase activity was improved. The results indicated that the reversible hydrogen-bond complexation between TOPO and caffeates significantly enhances the cooperative effect of the two phases on the lipase-catalyzed reaction. It increased the product stability, avoided the ability of oxidization and reduced the energy consumption. Compared with the packed bed microreactor [9], although the temperature was increased by 13 °C, the MC conversion and CAPE yield were both improved; the MC concentration of 10 g/L was 3.33-fold higher than that used before, so the overall cost is reduced greatly. Remarkably, the ratio of catalyst to donor was greatly decreased from 90:1 down to 20:1, reducing the dosage of immobilized lipase Novozym 435 to improve the technical feasibility in industry. Therefore, using the developed biphasic system to synthesize CAPE from MC and PE using enzymatic transesterification would be the most effective procedure to the best of our knowledge.

### 2.6. Antibacterial Activity of CAPE against R. solanacearum

**Figure 7:**
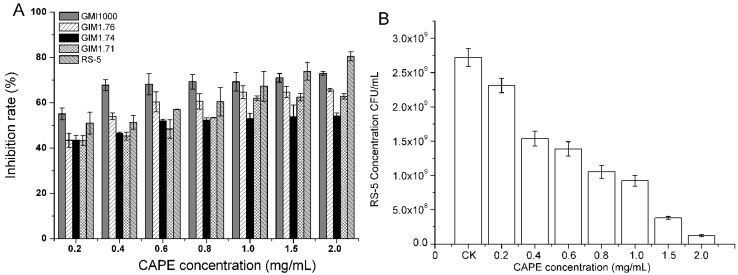
Bacterial growth inhibition (%) of CAPE against *R. solanacearum* strains including GMI1000, GIM1.76, GIM1.74, GIM1.71 and RS-5 measured at 600 nm (**A**), and the changes of mulberry bacterial wilt pathogen strain RS-5 treated by CAPE solutions with different concentrations tested by qPCR (**B**). Each value is expressed as the mean ± standard deviation (*n* = 3).

Figure 7A shows the growth status of five strains of *R. solanacearum* in the presence of CAPE at several concentrations. CAPE had a strong activity with the antibacterial rate over all 50% after 24 h treatment at a dosage of 2.0 mg/mL. Among five races, the RS-5 growth inhibition reached more than 80%, while it was only 54.12% for GIM1.74, and the others were inhibited from 62.83%–72.97%. The concentration of CAPE had little impact on GIM1.74, GIM1.71 and GMI1000. The results suggested that *R. solanacearum* was resistant towards CAPE, especially GIM1.74 and GIM1.71, probably due to the differences in the cell membrane structure of these bacterial strains, such as the intrinsic resistance characteristics to CAPE and the features of lipopolysaccharide molecules in the outer leaflet of their membranes [26,27]. Table 4 shows the half maximal effective concentration (EC_50_) values of CAPE against five *R. solanacearum* strains. The EC_50_ values of CAPE against *R. solanacearum* were all under 1.0 mg/mL, which suggested that these antibacterial efficiencies were obvious. Especially, the EC_50_ values against GMI1000, GIM1.76 and RS-5 were less than 0.3 mg/mL, so CAPE could be the most potent compound against *R. solanacearum*. In addition, for GIM1.74 and GIM 1.71, the EC_50_ values were both above 0.5 mg/mL, which suggested that the antibacterial efficiency was not readily evident. These results were well in accord with bacterial growth inhibition of CAPE. Thus, CAPE could be used as an in vitro inhibitor against GMI1000, GIM1.76 and RS-5.

Table 5 shows various agents for controlling bacterial wilt diseases caused by *R. solanacearum* in the present report. The control agents belong to two kinds, that is synthesized compounds and natural extracts or compounds. Among these synthesized compounds, 2-mercapto-5-substituted-1,3,4-oxadiazole/thiadiazole derivatives and imine derivatives of quinazolin-4(3*H*)-one have EC_50_ values against tomato bacterial wilts of 15.14–109.61 µg/mL [44] and 38.64–201.52 µg/mL [45], respectively. For natural extracts and compounds, the EC_50_ and MIC values of coumarin were 198.64 µg/mL and 384 µg/mL [46], and the MIC values of some plant extracts ranged from 3.91 µg/mL–128 µg/mL, including leaf essential oil isolated from *Macleaya cordata* R. Br. [47], yanglingmycin and its analogues [48] and polyphenols isolated from *Spiromastix* sp. MCCC 3A00308 [49]. Actually, the synthesized compounds might be harmful to the environment, and natural extracts or compounds also are unfeasible due to the costs for isolation. Our group prepared a series of chitosan (CTS)-*g*-CA using enzymatic modification of chitosan by cinnamic acids, with EC_50_ values of 42.1–800.0 µg/mL against *R. solanacearum* [32]. Compared with the above control agents, CAPE with EC_50_ values of 165.2–752.7 µg/mL is the most potent inhibitor against *R. solanacearum*.

To further evaluate the inhibitory effect of CAPE as an inhibitor against mulberry bacterial wilt pathogen strain RS-5, the reduced bacterial counts was determined by a quantitative real-time PCR (qPCR) method. Figure 7B shows the overnight changes of RS-5 treated by CAPE solutions with different concentrations. With an increase in the CAPE concentration from 0–2.0 mg/mL, the RS-5 concentration was obviously decreased from 2.72 × 10^9^ CFU/mL down to 1.23 × 10^8^ CFU/mL. The results indicated that the growth inhibition of RS-5 treated by CAPE presents a good concentration-response relationship, which also suggests that CAPE could be a potential in vitro inhibitor of plant pathogenic bacteria. Thus, we will deal with the limitation of its low water solubility to play a crucial role in many biological effects and bioavailability.

## 3. Materials and Methods

### 3.1. Materials and Strains

CA was purchased from Nanjing Zelang Pharmaceutical Sci. & Tech. Co. Ltd. (Nanjing, China), MC was synthesized according to the procedure described in our previous work [10,20,21]. CAPE standard and TOPO were purchased from Sigma Chemical Co. (St. Louis, MO, USA). Lipase Novozym 435 was purchased from Novozymes (Bagsvaerd, Denmark). [Bmim][Tf_2_N] was purchased from Shanghai Cheng-Jie Chemical Co., Ltd. (Shanghai, China). Methanol and acetonitrile were HPLC grade (Tedia Co., Fairfield, OH, USA), and all other reagents and solvents were analytical grade (Sinopharm Chemical Reagent Co., Ltd., Shanghai, China).

*R. solanacearum* strains contain Race 1 (GMI1000, purchased from the Guangdong Institute of Microbiology of Microbial Culture Collection Center (GIMCC)), Race 2 (GIM1.76, purchased from GIMCC), Race 3 (GIM1.74, donated by the Institute of Plant Protection, Nanjing Agricultural University), Race 4 (GIM1.71, purchased from GIMCC) and Race 5 (RS-5, isolated from diseased mulberry roots, among which RS-5 showed pathogenicity to mulberry after pathogenicity test). All strains were incubated aerobically at 30 °C in liquid casamino acid-peptone-glucose (CPG) medium (1 L distilled water with 10 g tryptone, 5 g glucose and 1 g casamino acids). Then, the bacterial solution was diluted with CPG medium to obtain a final optical density of OD_600_ = 0.1, which corresponded to ~10^8^ (CFU/mL). The final bacterial solution was used to measure the antibacterial activity in all experiments.

### 3.2. Selective Complexing Feasibility of TOPO and Caffeates in Cyclohexane/[Bmim][Tf_2_N]

Complexing experiments of TOPO and caffeates including MC and CAPE were performed at different temperature ranging from 20–60 °C. The Novozym 435-catalyzed synthesis reactant of CAPE from MC and PE in [Bmim][Tf_2_N] was used as the material [9]; TOPO and cyclohexane were used as the complexing agent and the diluent solvent. Cyclohexane containing a certain concentration of TOPO was used to extract target components during the lipase-catalyzed reaction. The solvent of 2 mL TOPO-cyclohexane and prepared reactant of 2 mL [Bmim][Tf_2_N] were added to 5-mL glass vials. Vials were agitated at 180 rpm for approximately 1 h using a mechanical shaker. The flasks were allowed to equilibrate and settle for 30 min before the two phases were separated. The IL and solvent phases were sampled and diluted for HPLC analysis, respectively.

To determine the feasibility of TOPO complexed with caffeates including MC and CAPE used in the biphasic system consisting of cyclohexane and [Bmim][Tf_2_N], the *D* value of caffeates during the reactive complexation was calculated using Equation (3):

(3) $$D=\frac{c_{\mathrm{org}}}{c_{\mathrm{IL}}}$$

where *c_org_* (g/L) is the equilibrium concentration of caffeates in the organic phase and *c*_IL_ is the equilibrium concentration of caffeates in the IL phase. *D*_CAPE_ is the *D* value of CAPE, and *D*_MC_ is the *D* value of MC. The β-value of CAPE vs. MC was calculated using Equation (4):

(4) $$\beta_{\text{CAPE},\;\text{MC}}=\frac{D_{\text{CAPE}}}{D_{\text{MC}}}$$

### 3.3. Lipase-Catalyzed Synthesis of CAPE from MC and PE via Transesterification Using the TOPO-Cyclohexane/[Bmim][Tf_2_N] System

The synthesis of CAPE from MC and PE was performed in a 5-mL screw-capped glass vial with a constant stirring speed of 180 rpm. TOPO-cyclohexane and [Bmim][Tf_2_N] were sequentially added to a vial to form the biphasic system, then MC and PE were added into it. The reaction was initiated after the addition of Novozym 435. An aliquot of each phase was taken for HPLC analysis at different time intervals. The effects of the TOPO concentration in cyclohexane, the mass ratio of MC to lipase, the reaction temperature, the molar ratio of MC to PE and the substrate concentration of MC on the CAPE yield were investigated, respectively. The optimum condition was optimized by RSM, and the corresponding methods and results are included in the Electronic Supporting Information.

### 3.4. Enzymatic Kinetics of CAPE Synthesis Using the Biphase System

The kinetics of transesterification in the batch reactor was investigated by studying the effects of the substrate concentrations on the initial rate of reaction. The concentrations of MC in the biphase and single phase varied within the ranges of 2–10 g/L and 1–5 g/L, respectively. Initial reaction rates, expressed as mM CAPE per minute and per gram of enzyme, were determined by the initial slope of a simulated second-order polynomial regression.

### 3.5. HPLC Analysis of Caffeates in the Enzymatic Synthesis of CAPE Using the Biphasic System

The concentrations of MC and CAPE were determined using an HPLC system equipped with a constant flow pump (2PB0540, Beijing Satellite Factory, Beijing, China) and a UV-Vis detector (L-7420, Techcomp Co. Ltd., Shanghai, China). Separation was performed on a Kromasil C_18_ column (250 mm × 4.6 mm, i.d.: 5 μm, Akzo Nobel Co., Amsterdam, The Netherlands) at 30 °C. The determinations of CAPE and MC were performed using acetonitrile/water (50:50, *v*/*v*) and methanol/water (65:35, *v*/*v*), respectively. The flow rate was 1 mL/min, and samples were detected under UV light at 325 nm. The CA conversion and CAPE yield of the lipase-catalyzed reaction were calculated as Equations (5) and (6), respectively [9,19].

(5) $$\text{CAPE}\;\text{yield}\;(\%)=\frac{\text{molar amount of CAPE}\;(\text{mol})}{\text{initial molar amount of MC}\;(\text{mol})}\times 100\%$$

(6) $$\text{MC}\;\text{Conversion}\;(\%)=\frac{\text{consumptive molar amount of MC}(\text{mol})}{\text{initial molar amount of MC}\;(\text{mol})}\times 100\%$$

### 3.6. ESI-LC-MS and ^1^H-NMR Analysis of Synthesis Product Using the Biphasic System

To confirm the product CAPE, reactants were injected into an LC-MS according to the following procedure: LC-MS analysis was performed on an analytical Kromasil column C_18_ (250 mm × 4.6 mm, i.d.: 5 μm, Akzo Nobel Co., Amsterdam, The Netherlands) using an Accela HPLC system and a TSQ Quantum Access tandem mass spectrometer (TSQ Quantum Access MAX, Thermo Fisher Scientific Inc., Boston, MA, USA) with an ESI source. The mass spectrometer was operated in the electron impact ionization mode, and selected ion monitoring (SIM) was employed under negative ion mode. The conditions were identical to previous studies [9,19], and the data were processed using Xcalibur 1.2 software (Figure S5).

According to the protocol reported [50], CAPE with a ca. 95% yield was obtained using preparative TLC (ethyl acetate/hexane, 5:1 *v*/*v*) from the synthetic product. ^1^H-NMR data of the product CAPE were acquired on a Bruker AVANCE spectrometer 400 (BrukerBiospin Co., Billerica, MA, USA) at room temperature. Five milligrams of the sample were dissolved in 0.5 mL of dimethylsulfoxide (DMSO)-*d*_6_, and chemical shifts were expressed in *δ* (ppm) values relative to tetramethylsilane (TMS) as an internal standard. The NMR data of caffeates were in accordance with previous studies [19], which are listed in the Electronic Supporting Information.

### 3.7. Characteristics of Lipase and Complex Formation Measured by FTIR Spectrometry

Novozym 435 was mixed in dimethyl sulfoxide (DMSO) with agitation at 37 °C for 30 min. The mixture was pumped and filtered, and the filter mass was washed by DMSO. The measurement of the Novozym 435 secondary structure was achieved using FTIR spectrometry measured by a Varian 670 IR spectrometer. All samples were overlaid on a zinc selenide attenuated total reflectance (ATR) accessory, and the spectrum was obtained from IR spectra. The secondary structure elements based on the information of the amide I region and the band assignment were manipulated using Omnic and Peakfit software [42]. Moreover, the complexes of TOPO and caffeates including MC and CAPE from the enzymatic reactant were prepared in the 30 g/L TOPO-cyclohexane/[Bmim][Tf_2_N] system, for which the formation characteristics were collected by IR spectra with a demountable BaF_2_ cell at 25 °C [24]. The spectrometer was purged with dry air. Conditions were 4 cm^−1^ spectral resolution, 20 kHz scan speed and 128 scan co-additions, and the region was 500–4000 cm^−1^.

### 3.8. Antibacterial Activity of CAPE against R. solanacearum

Antibacterial activity of CAPE was performed according to an improved protocol based on a previous method [32]. The CAPE solutions were prepared with Triton X-100 to obtain different concentrations. Ninety six-well cell culture plates were used to incubate the bacterial strains. To avoid cell death from nutritional deficiency, 140 μL of liquid CPG medium, 40 μL of diluted bacterial solution and 20 μL of different concentration samples (initial Triton X-100 was 100%, *v*/*v*, *T*_Sample_) were added into the wells in succession. In addition, one control was designed as: 40 μL of diluted bacterial solution and 140 μL of liquid CPG medium with 20 μL of Triton X-100 (100%, *v*/*v*, *T*_Blank_).

All plates were homogenized by constantly shaking, and after 10 min, the bacterial growth at T0 was determined by optical density at 600 nm on a microplate reader (SpectraMax i3, Silicon Valley, CA, USA) with a blank that consisted of liquid CPG medium. Then, the plates were incubated at 30 °C for 24 h. After incubation, the plates were shaken for 15 min to test for *TF*. The antibacterial activity was reported as an inhibition percentage (%) using Equation (7):

(7) $$\text{Inhibition}\;(\%)=(1-\frac{TF_{\text{sample}}-T0_{\text{sample}}}{TF_{\text{blank}}-T0_{\text{blank}}})\times 100\%$$

where *T0*_Sample_ and *TF*_Sample_ correspond to the optical density at 600 nm of the strain growth with the existence of the CAPE before (*T0*) and after (*TF*) incubation, respectively. Moreover, *T0*_Blank_ and *TF*_Blank_ correspond to the liquid CPG medium with Triton X-100 before and after incubation, respectively.

Further, to assay the reducing bacterial count of RS-5 treated by CAPE, a qPCR method based on a specific primer pair of RS-72F/RS-312R (RS-72F: 5′-ATGGATAAAGGGTTCGTGGTG-3′; RS-312R: 5′-CAGGCTCAGCGAGATTGC-3′) was developed. Briefly, 2 μL of each sample of overnight-cultured cells treated by different concentrations of CAPE/Triton X-100 with the specific primer and 18 μL mixed qPCR reagent (from TaKaRa, Bio, Japan) were added into the corresponding 96 × 0.2 mL PCR plates, then the reaction was generated. After centrifugation, the PCR plates were tested by a Fast Real-Time PCR System (ABI 7300) with a limit of detection of 50 pg/μL for DNA and 1.0 × 10^4^ CFU/mL for the cell suspension. *C*_0_ is the initial concentration of RS-5; the cell concentrations treated with CAPE were calculated by the standard curve of *C*_t_ value versus log numbers of cells from 2.68 × 10^4^–5.35 × 10^8^ CFU/mL as Equation (8):

(8) $$C_{t}=-2.71\mathrm{Log}C_{0}+39.89,\;R^{2}\;=0.9993$$

### 3.9. Statistical Analysis

The data were recorded as the mean ± standard deviation (SD). The EC_50_ values were fitted by logistic evaluation, and the 95% confidence levels were also determined. Statistical analysis was performed with the variance (ANOVA) method.

## 4. Conclusions

A biphase of TOPO-cyclohexane/[Bmim][Tf_2_N] (1:1, *v*/*v*) was firstly used in the Novozym 435-catalyzed synthesis of CAPE by the transesterification of MC with PE. Due to the selective complexing activity of TOPO to caffeates including MC and CAPE, the biphase had a more efficient productivity with a lesser cost. The MC conversion of 98.83% ± 0.76% and the CAPE yield of 96.29% ± 0.07% were obtained by RSM in the 25 g/L TOPO-cyclohexane/[Bmim][Tf_2_N] (1:1, *v*/*v*); the complex stoichiometry calculation and FTIR spectrum showed that the reversible hydrogen-bond complexation between TOPO and caffeates significantly enhances the cooperative effect of the two phases on the lipase-catalyzed reaction. The CAPE EC_50_ of 0.17–0.75 mg/mL against five *R. solanacearum* strains was firstly reported, suggesting that it is a potential inhibitor against bacterial wilt pathogen *R. solanacearum*.

## Figures and Tables

**Table 1:** IR peak changes of the characteristic groups in TOPO and the objectives after complex formation.

Molecular Target	Before (cm^−1^)	After (cm^−1^)	Δυ (cm^−1^)
P=O in TOPO changed by MC	1176	1157	−19
P=O in TOPO changed by CAPE	1176	1151	−25
–COO– in MC changed by TOPO	1683	1631	−52
–COO– in CAPE changed by TOPO	1684	1631	−53
–Ph–OH in MC changed by TOPO	3326	3210	−16
–Ph–OH in CAPE changed by TOPO	3326	3261	−65

**Table 2:** Kinetic constants of the lipase-catalyzed synthesis of CAPE under different media.

Medium	*K*_m(app)_ (mM)	R^2^
[Bmim][Tf_2_N] ^a^	1.46	0.9381
Cyclohexane/[Bmim][Tf_2_N] (1:1, *v*/*v*) ^b^	3.75	0.9986
30 g/L TOPO-cyclohexane/[Bmim][Tf_2_N] (1:1, *v*/*v*) ^b^	1.35	0.9310

^a^ Mass ratio of MC to Novozym 435 = 1:10, molar ratio of MC to PE = 1:20, temperature = 90 °C; ^b^ mass ratio of MC to Novozym 435 = 1:20, molar ratio of MC to PE = 1:20, temperature = 75 °C.

**Table 3:** Quantitative estimation (%) of the secondary structure elements of treated Novozym 435 calculated by FTIR analysis in the amide I region under different media.

Medium	α-Helix (%)				β-Sheet (%)			
	0 h	12 h	36 h	60 h	0 h	12 h	36 h	60 h
[Bmim][Tf_2_N] ^a^	12.8	65.8	0.0	19.0	55.2	21.5	18.3	36.0
Cyclohexane/[Bmim][Tf_2_N] (1:1, *v*/*v*) ^b^	12.8	12.4	9.4	6.1	55.2	4.8	0.0	0.0
30 g/L TOPO-cyclohexane/[Bmim][Tf_2_N] (1:1, *v*/*v*) ^c^	12.8	37.9	25.3	0.0	55.2	10.2	2.2	13.2

^a^ Mass ratio of MC to Novozym 435 = 1:10, MC concentration = 3 mg/mL, molar ratio of MC to PE = 1:20, temperature = 90 °C; ^b^ mass ratio of MC to Novozym 435 = 1:20, MC concentration = 3 mg/mL, molar ratio of MC to PE = 1:20, temperature = 75 °C; ^c^ mass ratio of MC to Novozym 435 = 1:20, MC concentration = 10 mg/mL, molar ratio of MC to PE = 1:20, temperature = 75 °C.

**Table 4:** Half maximal effective concentration (EC_50_) (mg/mL) values of CAPE against five *R. solanacearum* strains fitted by a logistic regression.

*R. solanacearum* Strains	EC_50_ (mg/mL)	95% Confidence Level
GMI1000	0.17	−0.162–0.178
GIM1.76	0.31	−0.542–0.322
GIM1.74	0.51	0.073–1.231
GIM1.71	0.75	0.349–0.808
RS-5	0.24	−0.05–0.323

**Table 5:** Various agents to control bacterial wilt diseases caused by *R. solanacearum* in the present report.

Compounds or Natural Extracts	Bacterial Strain	Inhibition Effect			References
		EC_50_ (µg/mL) ^a^	MIC (µg/mL) ^b^	DIZ (mm) ^c^	
2-Mercapto-5-substituted-1,3,4-oxadiazole/thiadiazole derivatives	*R. solanacearum*	15.14–109.61	-- ^d^	--	[44]
Imine derivatives of quinazoline-4(3*H*)-one	*R. solanacearum*	38.64–201.52	--	--	[45]
Coumarin	*R. solanacearum*	198.64	384	--	[46]
Leaf essential oil isolated from *Macleaya cordata* R. Br	*R. solanacearum*	--	125	18.6 ± 1.9	[47]
Yanglingmycin and its analogues	*R. solanacearum*	--	3.91–125	--	[48]
Polyphenols isolated from *Spiromastix* sp. MCCC 3A00308	*R. solanacearum*	--	0.5–128	--	[49]
CTS-*g*-CA	*R. solanacearum* including GMI1000, GIM1.76, GIM1.74, GIM1.71 and RS-5	42.1–800.0	--	--	[32]
CAPE	*R. solanacearum* including GMI1000, GIM1.76, GIM1.74, GIM1.71 and RS-5	165.2–752.7	--	--	This work

^a^ EC_50_, half maximal effective concentration; ^b^ DIZ, diameter of inhibition zone; ^c^ MIC, minimum inhibitory concentration; ^d^ not elsewhere specified.

## Supplementary Materials

The following are available online at http://www.mdpi.com/1420-3049/22/1/72/s1.
