# Supplementary material for: Cooperative Reinforcement of Ionic Liquid and Reactive Solvent on Enzymatic Synthesis of Caffeic Acid Phenethyl Ester as an In Vitro Inhibitor of Plant Pathogenic Bacteria
Source: Molecules. 2017 Jan 2;22(1):72. doi: 10.3390/molecules22010072 (PMC6155643; doi:10.3390/molecules22010072)
Supplement: Supplementary file 1 [file molecules-22-00072-s001.pdf]

Supplementary Materials: Cooperative Reinforcement of Ionic Liquid and Reactive Solvent on Enzymatic Synthesis of Caffeic Acid Phenethyl Ester as an *In Vitro* Inhibitor of Plant Pathogenic Bacteria

Yan Xu, Sheng Sheng, Xi Liu, Chao Wang, Wei Xiao, Jun Wang and Fu-An Wu

1. Methods

1.1. Experimental Design for Response Surface Methodology (RSM)

A Box–Behnken design (BBD) was employed to obtain the optimum biphase reaction conditions for the enzymatic synthesis of CAPE using the transesterification of MC with PE in a TOPO-cyclohexane/ionic liquid system, for which the MC concentration was 10 mg/mL, and the molar ratio of PE to MC was 20:1. A 3-level 3-factor BBD was adopted in this study, requiring 17 experiments. The parameters, which were selected for the study of CAPE synthesis, and their respective levels were as follows: reaction temperature (65–85 °C), TOPO concentration (10–50 mg/mL), reaction time (48–72 h). Table S1 shows the uncoded independent factors, levels and experimental design.

Table S1. The 3-level 3-factor Box–Behnken design and response surface analysis of the experimental CAPE yields using a TOPO-cyclohexane/[Bmim][Tf_2N] (1:1, *v*/*v*) system.

Std.	Run	Temperature (°C)	TOPO Concentration (g/L)	Time (h)	CAPE Yield (%)	
					Observed	Predicted
1	4	75	10	48	77.93	75.82
2	12	75	30	60	91.64	93.66
3	13	75	50	48	55.15	53.04
4	6	65	50	60	43.39	41.97
5	16	75	30	60	93.64	93.66
6	3	75	10	72	68.95	71.06
7	15	75	30	60	95.65	93.66
8	14	75	30	60	95.78	93.66
9	11	85	30	72	73.97	70.44
10	8	85	10	60	70.01	71.43
11	5	85	50	60	55.44	56.86
12	2	75	50	72	50.9	53.01
13	9	65	10	60	69.64	68.22
14	7	75	30	60	91.59	93.66
15	10	65	30	48	60.24	63.77
16	17	65	30	72	60.63	59.94
17	1	85	30	48	70.70	71.39

1.2. Spherical Resin and Lipase Analysis by FTIR Spectrometry

Novozym 435 was mixed in dimethyl sulfoxide (DMSO) with agitation at 37 °C for 30 min. The mixture was pumped and filtered, and the filter mass was washed by DMSO. Spherical recycled resin was soaked by 5% Triton X-100 with agitation at 37 °C for 30 min; the mixture was pumped and filtered, and the filter mass was successively washed by 5% Triton X-100 and distilled water. Then, the resin without protein was dried at 55 °C for 24 h. The measurement was achieved using FTIR spectrometry (Varian 670). IR spectra were measured at 25 °C. Conditions were 4 cm^{-1} spectral

resolution, 20 kHz scan speed and 128 scan co-additions, and the region was 500–4000 cm^{-1}. Spherical resin and lipases were overlaid on a zinc selenide attenuated total reflectance (ATR) accessory, and the spectrum was obtained from the IR spectra [1].

1.3. LC-MS and 1H-NMR analysis of Products

To confirm the product CAPE using Novozym 435-catalyzed transesterification in a 30 g/L TOPO-cyclohexane/[Bmim][Tf_2N] (1:1, *v*/*v*) system, reaction mixtures were injected into an LC-MS. The experimental conditions were identical to those employed in previous studies [2]. The data were processed using Xcalibur 1.2 software. The CAPE product (5 mg) was dissolved in 0.5 mL of DMSO-d_6. ^{1}H-NMR spectra were recorded on a Bruker AVANCE 400 instrument operated at a ^{1}H resonance frequency of 400 MHz [3].

2. Results and Discussion

2.1. Experimental Design for RSM

To systemically explore the relationships between the effect of reaction temperature, TOPO concentration and reaction time on the CAPE yield, a three-level three-factor BBD was applied. The ANOVA for the response surface quadratic model is provided in Table S2.

Table S2. Analysis of variance (ANOVA) for the experimental design in the TOPO-cyclohexane/[Bmim][Tf_2N] (1:1, *v*/*v*) system.

Source	Sum of Squares	DF	Mean Square	*F*-Value	*p*-Value
Model	4442.88	9	493.65	50.32	<0.0001
Residual	68.67	7	9.81		
Lack of fit	51.85	3	17.28	4.11	0.1028
Pure error	16.82	4	4.21		
Corresponding total	4511.55	16			
R^2	0.9848				
Adjusted R^2	0.9652				

According to the statistical method, a quadratic regression model for CAPE yield was obtained using coded values, which are provided in Equation (S1):

$$Y (\%) = 93.66 + 4.53A - 10.21B - 1.20C + 2.92AB + 0.72AC + 1.18BC - 15.44A^2 - 18.60B^2 - 11.83C^2 \quad \text{(S1)}$$

where Y is the response factor CAPE yield (%), and A, B and C are the values of the independent factors temperature (°C), TOPO concentration (mg/mL) and time, respectively.

For the fitted model, the coefficients of the above equation were estimated at a 95% confidence level. The coefficient of determination R^2 was 0.9848. Based on the results of the *F*-test, the regression quadratic model was highly significant ($p < 0.0001$), and the lack of fit was insignificant ($p > 0.05$). These statistical tests indicated that the model adequately described the relationship between reaction parameters and the CAPE yield.

The statistical analysis of the experimental data indicated that the mutual effects between temperature and TOPO concentration were significant. Figure S1A demonstrates the surface plot of the response for the CAPE yield. The 3D response surface revealed that incremental increases in TOPO concentrations from 10 g/L–30 g/L led to an increase in CAPE yield with increased temperature. On the contrary, an increase in temperature did not significantly improve the CAPE yield at low TOPO concentrations. Figure S1B shows the effects of temperature and time on CAPE yield. For a short time, increasing the temperature greatly improved the CAPE yield. In addition, Figure S1C shows the effects of the interaction between TOPO concentrations and time on CAPE yield; increasing this value had a negative effect for a long time and high TOPO concentrations. At

the lowest temperature (65 °C) and the highest TOPO concentration (50 mg/mL), the CAPE yield was only 43.39%. At a modest temperature (75 °C) and modest TOPO concentration (30 g/L), the highest CAPE yield was 93.66%.

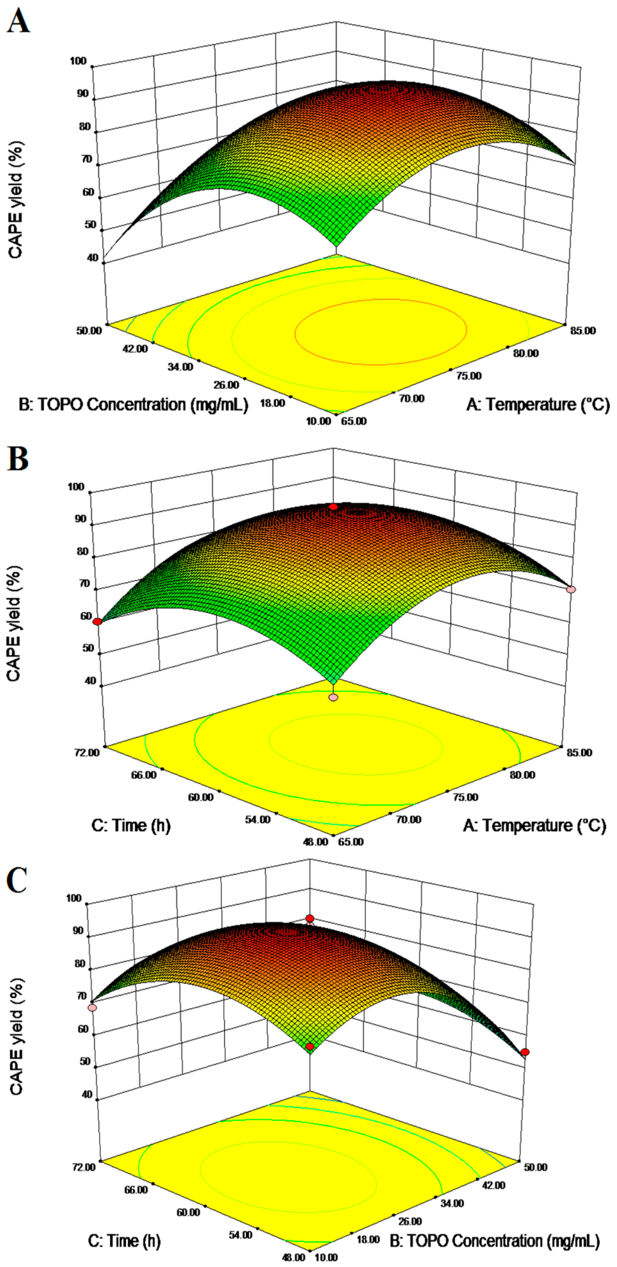

Figure S1. Response surface and contour diagrams of CAPE yield in the TOPO-cyclohexane/[Bmim][Tf2N] (1:1, *v*/*v*) system as a function of (**A**) temperature and TOPO concentration interaction on CAPE yield; (**B**) temperature and time interaction on CAPE yield; and (**C**) TOPO concentration and time interaction on CAPE yield.

The optimal values of the selected variables were determined by ridge max analysis, which was obtained using Design-Expert 8 software. The optimal conditions for CAPE synthesis estimated by the model equation were as follows: temperature of 76 °C, TOPO concentration of 25 g/L and time of 59 h. The theoretical CAPE yield under the above conditions was 95.33%. To verify the adequacy of the prediction model, the optimal reaction conditions were carried out for CAPE synthesis in three independent replicates. The MC conversion of 98.83% ± 0.76% and CAPE yield of 96.29% ± 0.07% were obtained, a figure well within the estimated value of the model equation. This experiment confirmed the validity and adequacy of the predicted models.

2.2. Enzymatic Kinetics

Under the optimal conditions for CAPE synthesis, the enzymatic kinetics parameters were calculated. Figure S2 shows the Lineweaver–Burk plots of the lipase-catalyzed synthesis of CAPE via transesterification under different media.

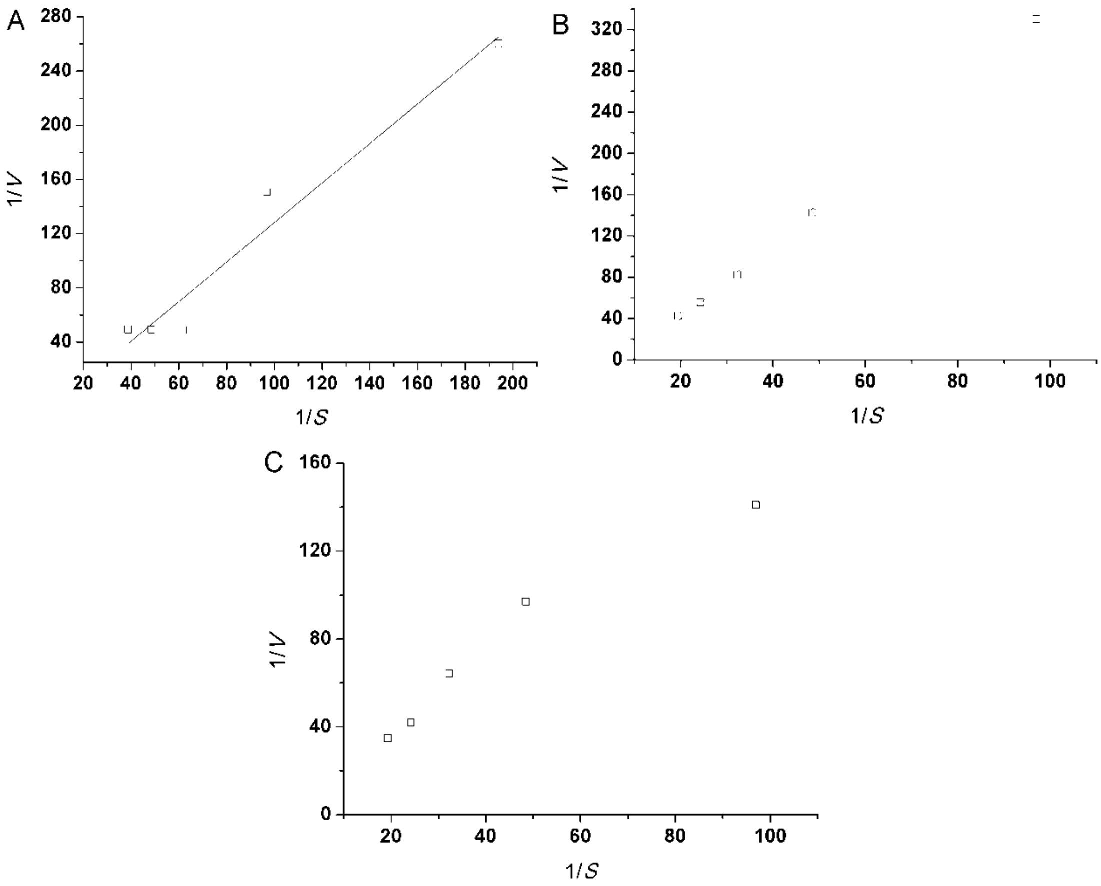

Figure S2. The Lineweaver–Burk plots of the lipase-catalyzed synthesis of CAPE via transesterification under different medium. (**A**) [Bmim][Tf_2N], conditions: mass ratio of MC to Novozym 435 = 1:10, molar ratio of MC to PE = 1:20, temperature = 90 °C; (**B**) cyclohexane/[Bmim][Tf_2N] (1:1, *v*/*v*), conditions: mass ratio of MC to Novozym 435 = 1:20, molar ratio of MC to PE = 1:20, temperature = 75 °C; (**C**) 30 g/L TOPO-cyclohexane/[Bmim][Tf_2N] (1:1, *v*/*v*), conditions: mass ratio of MC to Novozym 435 = 1:20, molar ratio of MC to PE = 1:20, temperature = 75 °C.

2.3. Lipase and Spherical Resin of Novozym 435 Analysis by FTIR Spectrometry

Figures S3 and S4 show the infrared absorption spectrum of carrier resin and lipase from Novozym 435 analyzed by FTIR spectrometry, respectively. The secondary structure of Novozym 435 suspended in three different media for 0 h, 12 h, 36 h and 60 h was analyzed to assist with explaining the lipase stability.

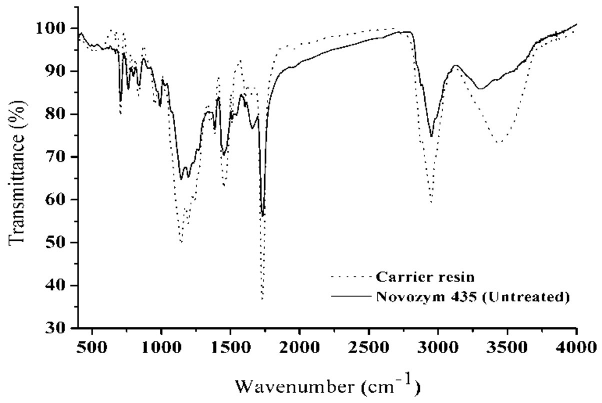

Figure S3. Infrared absorption spectrum of carrier resin and media untreated Novozym 435.

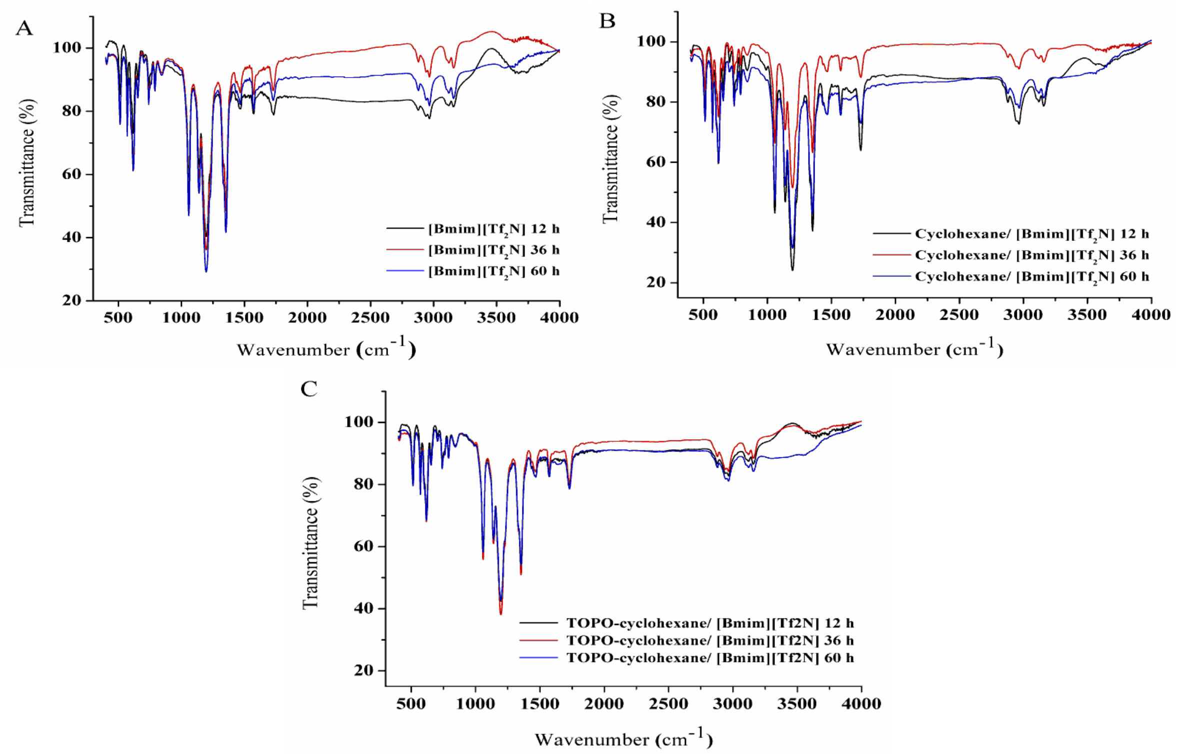

Figure S4. Infrared absorption spectrum of lipase extracted from Novozym 435 under different media analyzed by FTIR spectrometry. (**A**) [Bmim][Tf_2N], conditions: mass ratio of MC to Novozym 435 = 1:10, MC concentration = 3 mg/mL, molar ratio of MC to PE = 1:20, temperature = 90 °C; (**B**) cyclohexane/[Bmim][Tf_2N] (1:1, *v*/*v*), conditions: mass ratio of MC to Novozym 435 = 1:20, MC concentration = 10 mg/mL, molar ratio of MC to PE = 1:20, temperature = 75 °C; (**C**) 30 g/L TOPO-cyclohexane/[Bmim][Tf_2N] (1:1, *v*/*v*), conditions: mass ratio of MC to Novozym 435 = 1:20, MC concentration = 10 mg/mL, molar ratio of MC to PE = 1:20, temperature = 75 °C.

2.4. Confirmation of Products by LC-MS and NMR

Figure S5 shows that LC-MS confirmation of MC and CAPE from the lipase-catalyzed synthesis product of MC and PE in a 30 g/L TOPO-cyclohexane/[Bmim][Tf_2N] (1:1, *v*/*v*) system. The intense peaks at *m*/*z* 193.1 and *m*/*z* 283.1 in the ESI-MS spectra under negative ion mode corresponded to the deprotonated $[M - H]^-$ ions of MC and CAPE, respectively.

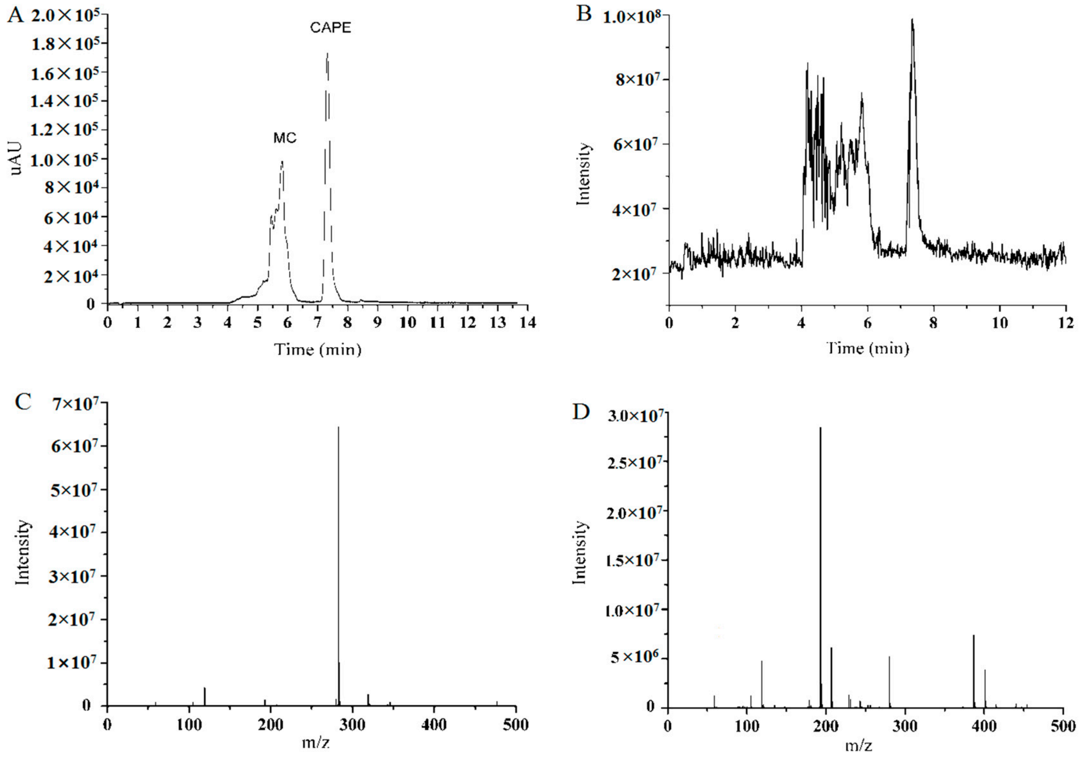

Figure S5. LC-MS confirmation of MC and CAPE from the lipase-catalyzed synthesis product of MC and PE using a 30 g/L TOPO-cyclohexane/[Bmim][Tf_2N] (1:1, *v*/*v*) system. (**A**) LC-UV chromatography of the reaction product; (**B**) LC-TIC chromatography of the reaction product; (**C**) MS spectrum of CAPE (*m*/*z* ratio = 283.08); (**D**) MS spectrum of MC (*m*/*z* ratio = 193.04).

The chemical shifts were reported relative to tetramethylsilane as the internal standard. The ^{1}H-NMR data (400 MHz, DMSO-d_6) of CAPE were as follows: δ 9.62 (s, 1H), 9.15 (s, 1H), 7.44 (d, *J* =15.9 Hz, 1H), 7.34–7.25 (m, 4H), 7.22 (ddd, *J* =5.7, 4.2, 1.9 Hz, 1H), 7.04 (d, *J* = 2.0 Hz, 1H), 6.99 (dd, *J* = 8.2, 2.0 Hz, 1H), 6.76 (d, *J* = 8.1 Hz, 1H), 6.22 (d, *J* = 15.9 Hz, 1H), 4.32 (t, *J* = 6.9 Hz, 2H), 2.95 (t, *J* =6.9 Hz, 2H). The LC–MS and NMR data for CAPE were in accordance with those in the literature [3].

References

1. Qin, J.; Zou, X.; Lv, S.; Jin, Q.; Wang, X. Influence of ionic liquids on lipase activity and stability in alcoholysis reactions. *RSC Adv.* **2016**, *6*, 87703–87709.
2. Wang, J.; Li, J.; Zhang, L.X.; Gu, S.S.; Wu, F.A. Lipase-catalyzed synthesis of caffeic acid phenethyl ester in ionic liquids: Effect of specific ions and reaction parameters. *Chin. J. Chem. Eng.* **2013**, *21*, 1376–1385.
3. Gu, S.S.; Wang, J.; Wei, X.B.; Cui, H.S.; Wu, X.Y.; Wu, F.A. Enhancement of lipase-catalyzed synthesis of caffeic acid phenethyl ester in ionic liquid with DMSO co-solvent. *Chin. J. Chem. Eng.* **2014**, *22*, 1314–1321
